# Analysis of Mechanical Properties and Failure Mechanism of Lightweight Aggregate Concrete Based on Meso Level

**DOI:** 10.3390/ma16155283

**Published:** 2023-07-27

**Authors:** Safwan Al-sayed, Xi Wang, Yijiang Peng

**Affiliations:** The Key Laboratory of Urban Security and Disaster Engineering, Ministry of Education, Beijing University of Technology, Beijing 100124, China; alsayed.safwan@yahoo.com

**Keywords:** lightweight aggregate concrete, mesoscopic, Delaunay triangular, base force element method (BFEM)

## Abstract

The relationship between the macroscopic mechanical properties of lightweight aggregate concrete and its microstructure is a hot topic in the discipline of concrete materials. It is very meaningful to provide an efficient numerical analysis method to conduct a meso-level analysis. This study proposes an automatic dissection algorithm and adapts the calculation program of the base force element method to conduct a non-linear damage analysis. In the numerical simulation, three groups of 100 mm × 100 mm × 100 mm specimens were selected for the uniaxial compression experiment and uniaxial tensile experiment, respectively. The average tensile strength of the numerical simulation for the uniaxial compression test was 21.86 MPa. The stress–strain softening curve, stress contour plot, strain contour plot, and damage plot of the light aggregate concrete were also analyzed. These research results provide data for analyzing the failure mechanism of light aggregate concrete and reveal the failure mechanism of light aggregate concrete. At the same time, the reliability of the proposed algorithm is verified. Our aim is to provide a more efficient and accurate analysis of meso-damage in lightweight aggregate concrete, which benefits industries involved in production, construction, and structural engineering.

## 1. Introduction

Lightweight aggregate concrete, especially artificial lightweight aggregate concrete, has the characteristics of being lightweight and multi-functional as well as having a high level of strength. It is a green energy-saving building material as well as a wall material for the promotion and application of prefabricated buildings. At present, the mechanical properties and damage mechanism of lightweight aggregate concrete are hot topics in the engineering field, and the relationship between its meso-structure and macroscopic mechanical properties is also a cutting-edge research topic in the academic community [1]. Many scholars have studied the mechanical properties and damage modes of lightweight aggregate concrete by controlling experimental variables to measure the relevant mechanical parameters of lightweight aggregate concrete, followed by in-depth and detailed research and analyses [2,3]. For instance, in an experimental work, Guo et al. [4] investigated the compressive strength and deformation behavior of basic magnesium sulfate cement–coral aggregate concrete (BMSC–CAC) through experiments and a 3D mesoscopic analysis. Their study provided insights into the mechanical properties of BMSC–CAC for the design of sustainable concrete structures. Wu et al. [5] presented an experimental and mesoscopic modelling investigation of the dynamic compressive behaviors of a new carbon-fiber-reinforced cement-based composite. Their study provided insights into the mechanical properties of the composite material under dynamic loading conditions. Wang et al. [6] investigated the influence of nano-silica on the performance of concrete using multi-scale experiments and micro-scale numerical simulations. Their study aimed to understand the mechanism behind nano-silica’s effect on the properties of concrete, providing insights for optimizing the use of nano-silica in concrete applications. Ma et al. [7] investigated the uniaxial compressive properties of ecological concrete using an experimental and three-dimensional (3D) mesoscopic analysis. Their study aimed to provide insights into the mechanical properties of ecological concrete and guide its practical application in sustainable construction. Wang et al. [8] conducted experiments and meso-scale numerical simulations to explore how the incorporation of ceramsite could enhance the compressive properties of foam concrete. Their study provided insights into the mechanical properties of the composite material and its potential applications in building construction. Ma et al. [9] investigated the behavior of coral aggregate concrete under dynamic splitting tensile loading using an experimental and three-dimensional mesoscopic analysis. Their study provided insights into the mechanical properties of the material and its performance under dynamic loading conditions, which can guide its practical application in engineering structures.

In addition, numerical analyses and analysis methods for lightweight aggregate concrete and other concretes have also been studied. For examples, Chen et al. [10] analyzed the remaining mechanical characteristics of the material by subjecting recycled pebble aggregate concrete to elevated temperatures and subsequently cooling it using a fire hydrant. Du et al. [11] examined the behavior of carbon-fiber-reinforced polymer (CFRP) confined rectangular concrete-filled steel tube (CFT) columns under axial compression, utilizing high-strength materials. Their investigation incorporated a numerical analysis and introduced a carrying capacity model. Their findings indicated that the proposed model successfully predicted the carrying capacity of CFRP confined rectangular CFT columns with high-strength materials when subjected to axial compression. Huang and Hu [12] provided a detailed examination at the meso-scale level, studying the failure characteristics and mechanical properties of lightweight aggregate concrete (LWAC) when subjected to axial tension. The LWAC samples were specifically analyzed with varying aggregate volume fractions and shapes. Their study aimed to understand the effect of the aggregate volume fraction and shape on the mechanical behavior of LWAC. Liu et al. [13] presented a numerical investigation into the size effect of LWAC on its tensile strength. Their study aimed to understand how the size of the specimen affects the tensile strength of LWAC and to identify the critical size beyond which the strength decreases significantly. Mačiūnas et al. [14] conducted a numerical simulation to investigate the thermal conductivity and thermal stress in lightweight refractory concrete containing cenospheres. Their study aimed to understand how the addition of cenospheres affects the thermal conductivity and thermal stress of the refractory concrete and to optimize its performance for high-temperature applications. Ren et al. [15] presented a numerical investigation into the dynamic increase factor (DIF) of ultra-high-performance concrete (UHPC) based on the split-Hopkinson pressure bar (SHPB) technology. Their study aimed to understand the dynamic mechanical behavior of UHPC and to evaluate the DIF, which is an important parameter for designing structures subjected to dynamic loads. Wang et al. [16] presented a study on the bond-slip behavior of LWAC using a virtual crack model with exponential softening characteristics. Their study aimed to understand the bond-slip behavior between the reinforcing steel bar and LWAC and to develop a reliable numerical model to predict it.

The meso-damage analysis of many concrete materials is mostly carried out by conventional finite element software. The research of different analytical methods needs further exploration. The base force element method (BFEM) utilizes either the potential energy principle or the complementary energy principle as its foundation and employs base force elements to represent the behavior of intricate structures [17,18,19]. This new method has been shown to be effective, efficient, and accurate in predicting the behavior of complex systems, making it a valuable tool for engineers and scientists. Wang et al. [17] introduced a numerical model employing a degenerate element of the BFEM to simulate the interfacial transition zone (ITZ) in recycled aggregate concrete (RAC). Their model considered the influence of the ITZ on the mechanical properties of RAC, enabling the optimization of RAC structure designs. Their study showed that their proposed model can effectively simulate the ITZ behavior in RAC and provide insight into the mechanical behavior of RAC structures. Wang et al. [18] introduced a novel technique called the base force element method, which utilizes the complementary energy principle for assessing structural damage in recycled aggregate concrete constructions. This method was shown to be effective in predicting the failure behavior of the structures under different loading conditions and levels of damage. Peng et al. [19] applied the BFEM and digital image processing (DIP) technology to manage the digital images of a recycled concrete cross-section. Significant information on the five-phase material of recycled aggregate concrete (RAC) was obtained through ternary processing, median filtering, and boundary processing. Their results showed that the five-phase material information agreed well with the real image, and it verified the applicability of the processing method. According to the extracted five-phase RAC data, the real aggregate distribution model based on the BFEM was established, and the uniaxial compression and uniaxial tension of the recycled concrete specimen were simulated.

Moreover, when it comes to the discussion of the methods of fracture mechanics at the meso level, the challenges associated with using recycled aggregates in concrete construction were addressed by proposing a novel equation to predict the shear strength of recycled aggregate concrete (RAC) Z push-off specimens. The aim of the study was to provide a reliable method for determining the shear strength of RAC structures. The proposed equation was developed based on experimental testing and an analysis, demonstrating improved accuracy compared to that of existing methods. This research enhances our understanding and practical applications of recycled aggregate concrete in structural engineering [20]. In a separate study, a straightforward method for determining the true specific fracture energy of concrete was introduced by Karihaloo et al. [21,22]. The importance of assessing the toughness and durability of concrete structures was emphasized, and a novel experimental procedure involving direct tension tests on notched concrete specimens was presented. A simplified approach to calculate the true specific fracture energy was proposed. The simplicity and effectiveness of the method in accurately determining fracture energy values were highlighted. These advancements in our understanding of fracture mechanics and ability to predict shear strength offer valuable insights for researchers and engineers involved in concrete material characterization and structural design.

In this study, a novel approach using the BFEM to analyze meso-damage in lightweight aggregate concrete is introduced. The objective of this study is to develop a random aggregate model for lightweight aggregate concrete and utilize the Delaunay triangulation method to create an algorithm for automatically subdividing layered meshes within the lightweight aggregate. Additionally, this study aims to develop a program for the automatic subdivision of lightweight aggregate concrete and adapt the calculation program of the base force element method to conduct a non-linear damage analysis to investigate the mechanical characteristics and failure mechanisms of this concrete variety. The significance of this study to related industries is noteworthy. By proposing an automatic dissection algorithm based on the Delaunay triangular mesh algorithm, this study offers an efficient solution for pre-processing in finite element calculations. This advancement can significantly improve the efficiency and accuracy of analyzing lightweight aggregate concrete, providing valuable insights into its mechanical behavior and failure mechanisms. The findings from this study can have practical implications for industries involved in lightweight aggregate concrete production, construction, and structural engineering, enabling them to optimize material properties, enhance structural designs, and ensure the durability and safety of concrete structures.

## 2. Stiffness Matrix of a Triangular Element by the BFEM

**Figure 1:**
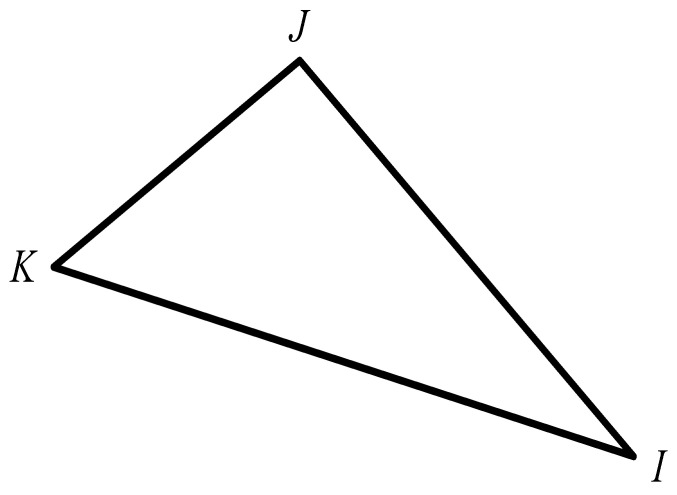
Construction of the stiffness of a triangular element.

In a Cartesian coordinate system, when a plane strain problem occurs, the stiffness matrix $K^{IJ}$ of a triangular element can be represented as Equation (1) [19]:

(1) $$K^{IJ}=\frac{E}{2A(1+v)}[\frac{2\nu }{1-2\nu }(m_{x}^{I}m_{x}^{J}e_{x}⊗e_{x}+m_{x}^{I}m_{y}^{J}e_{x}⊗e_{y}+m_{y}^{I}m_{x}^{J}e_{y}⊗e_{x}+m_{y}^{I}m_{y}^{J}e_{y}⊗e_{y})\;+\left(m_{x}^{I}m_{x}^{J}e_{x}⊗e_{x}+m_{x}^{I}m_{y}^{J}e_{y}⊗e_{x}+m_{y}^{I}m_{x}^{J}e_{x}⊗e_{y}+m_{y}^{I}m_{y}^{J}e_{y}⊗e_{y}\right)]$$

where I,J(I,J=1,2,3) is the local code of the element node, and $m_{x}^{I}$, $m_{y}^{I}$,${\;m}_{x}^{J}$, and $m_{y}^{J}$ are as shown in Figure 1, which can be expressed as Equations (2) and (3) [19]:

(2) $$\left\{\begin{array}{c} m_{x}^{I}\\ m_{y}^{I} \end{array}\right\}=\frac{1}{2}\left\{\begin{array}{c} y_{J}-y_{K}\\ x_{K}-x_{J} \end{array}\right\}$$

(3) $$\left\{\begin{array}{c} m_{x}^{J}\\ m_{y}^{J} \end{array}\right\}=\frac{1}{2}\left\{\begin{array}{c} y_{K}-y_{I}\\ x_{I}-x_{K} \end{array}\right\}$$

**Figure 2:**
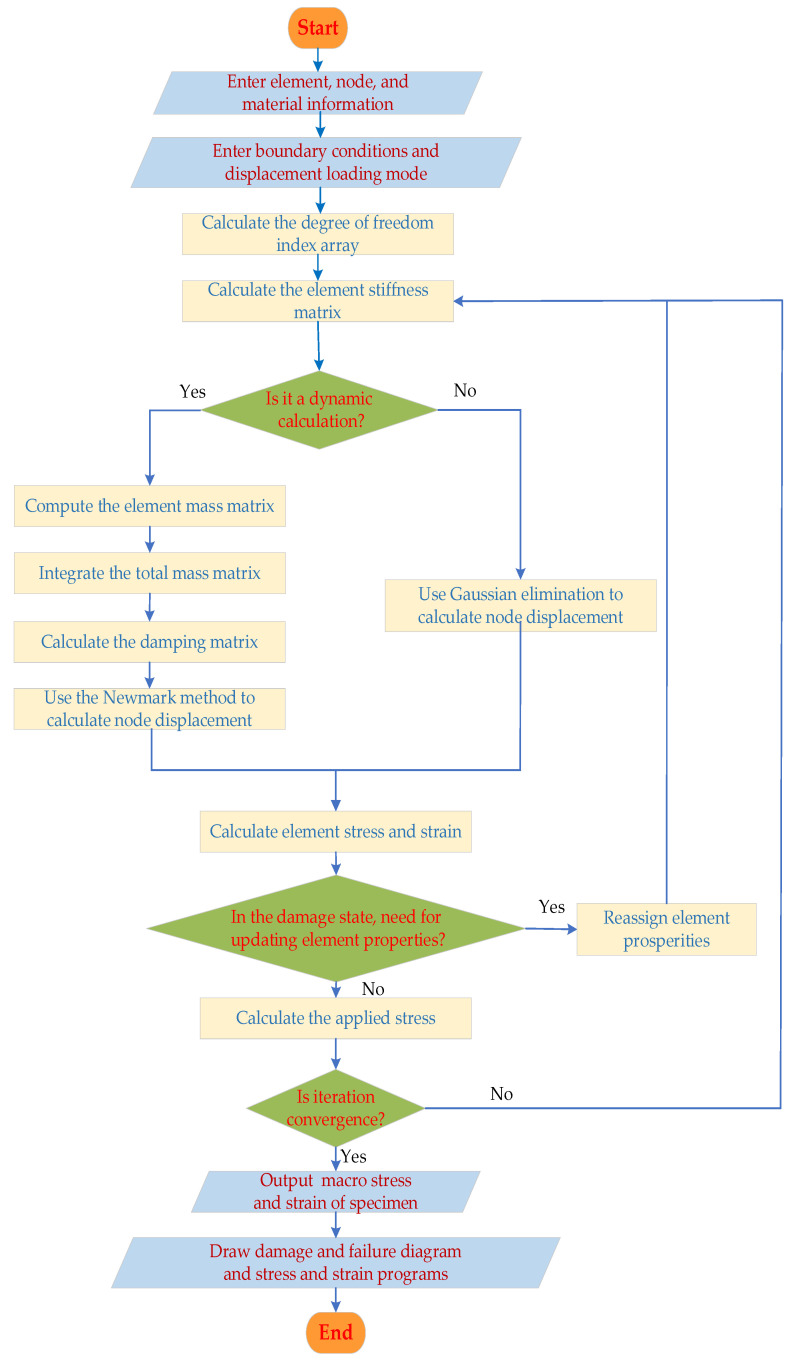
The main program flow chart of the BFEM.

The main program algorithm for the two-dimensional potential energy method using the basic element force method is shown in Figure 2.

## 3. Mesoscopic Numerical Model of Randomly Distributed Aggregates

### 3.1. Aggregate Number and Grading of Lightweight Aggregates

In this study, circular shapes were used to simulate lightweight aggregates in the numerical specimens of lightweight aggregate concrete. The grading curve of aggregates refers to a smooth curve plotted with the maximum size of sieves of different sizes through which particles with different particle sizes in the aggregate pass. In this study, the lightweight aggregates were all modeled using Fuller’s maximum density curve, also known as the Fuller grading curve, which can be used to describe the particle size distribution of aggregates, as expressed in Equation (4) [23].

(4) $$P\left(D<D_{0}\right)=100\%\times {\left(\frac{D_{0}}{D_{\max}}\right)}^{0.5}$$

where $D_{max}$ represents the diameter of the sieve; *D*_max_ represents the maximum particle size of the aggregate; and $P(D<D_{0})$ represents the mass percentage of the aggregate particles smaller than $D_{0}$. Walraven [23] and others established a conversion equation between the three-dimensional aggregate volume content of concrete specimens and the two-dimensional aggregate area content in any cross-section of the specimen based on the Fuller curve. When $D<D_{0}$, the probability of the aggregate particle size in the two-dimensional cross-section of the specimen is as in Equation (5) [23]:

(5) $$\begin{array}{l} P_{c}\left(D<D_{0}\right)=P_{k}[1.065{\left(\frac{D_{0}}{D_{\max}}\right)}^{0.5}-0.053{\left(\frac{D_{0}}{D_{\max}}\right)}^{4}-0.012{\left(\frac{D_{0}}{D_{\max}}\right)}^{6}\\ \;\;\;\;\;\;\;\;\;\;\;\;\;\;\;\;\;\;-0.0045{\left(\frac{D_{0}}{D_{\max}}\right)}^{8}+0.0025{\left(\frac{D_{0}}{D_{\max}}\right)}^{10}] \end{array}$$

where $P_{c}$ represents the probability that the aggregate particle size at any position in the section of the specimen is less than $D_{0}$; $P_{k}$ represents the ratio of the volume of aggregate particles smaller than $D_{0}$ to the total volume of concrete specimens; and $D_{max}$ represents the maximum particle size of the aggregate. Then, the number *n* of aggregate particles with sizes $D_{1}<D<D_{2}$ within different cross-sectional areas can be obtained as shown in Equation (6).

(6) $$n=\left[P_{c}\left(D<D_{2}\right)-P_{c}(D<D_{1})\right]\times A/A_{i}$$

where A represents the cross-sectional area of the lightweight aggregate concrete specimen, and $A_{i}$ represents the area of the aggregate that represents the particle size.

### 3.2. Distribution of Lightweight Aggregates

A set of random numbers was generated using FORTRAN programming to determine the coordinates of the lightweight aggregates. In this study, the Monte Carlo method was used, and the program generated several sets of pseudorandom numbers using the multiply-add congruential method [18].

(7) $$\left\{ \begin{array}{l} X_{n+1}=(\lambda X_{n}+C)(\mathrm{mod}M)\\ \xi_{n+1}=X_{n+1}/M \end{array}\right.$$

Equation (7) represents a recursive equation used for generating pseudo-random numbers uniformly distributed within the (0,1) range, where $X_{1}$ is an arbitrarily selected initial value, and the equation uses the multiply-with-carry method. The values λ=2053 and C=13,849 are then chosen. In order to facilitate computer calculations, the parameter *M* is calculated according to Equation (8) [18].

(8) $$M=2^{S}$$

in which S is the maximum possible number of effective binary digits in the computer, which is set to 16 in this study. The steps for placing the lightweight aggregate are as follows:

i. Determine the boundary and coordinate system based on the known dimensions of the lightweight aggregate concrete specimen.
ii. Utilize the Monte Carlo method to acquire random numbers *R_n_* and *E_n_* of the nth aggregate both falling within the range of (0,1), following a uniform distribution.
iii. Use these two random numbers to determine the coordinates for placing the aggregate $\left(X_{n},y_{n}\right)$ as shown in Equation (9).

(9) $$\left\{ \begin{array}{l} x_{n}=R_{n}\times b\\ y_{n}=E_{n}\times h \end{array}\right.$$

where *b* is the width of the specimen section, and *h* is the height of the specimen section.

According to the obtained coordinates, the bone center coordinates are used for the bone placement, ensuring that the lightweight aggregate and its surrounding old mortar and interface are within the range of the specimen’s side length. In order to prevent bone overlap, the distance between adjacent bone center points is set to be greater than 1.2 times the sum of the two bone diameters.

### 3.3. Finite Element Meshing of Numerical Specimen Model

When establishing a refined numerical model of concrete material, finite element meshing of the specimen is the first step. When using digital image technology for modeling, the mesh can be divided based on pixel points [19]. However, when using a random aggregate model or other parametric modeling methods, the triangulation method is widely employed as a means of creating finite element meshes, making it a commonly utilized approach. This study proposes two different triangulation methods and applies these methods to establish a meso-structure model of lightweight aggregate concrete.

#### 3.3.1. Mesh Model of Uniform Grid Generation

**Figure 3:**
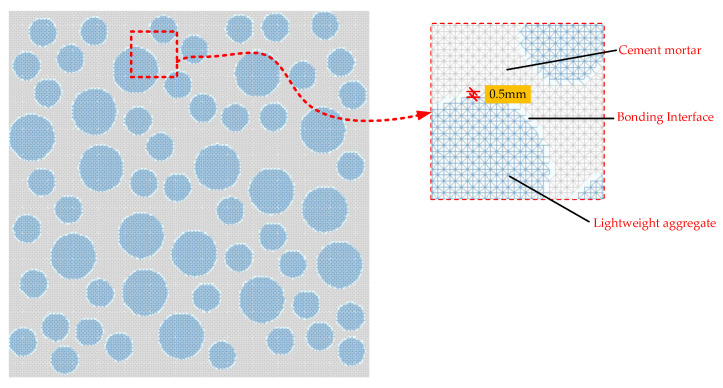
Uniform mesh projection model (Mesh generation method 1).

Referring to the method of Peng et al. [19], this study uses Fortran language to write a program that generates uniformly sized element nodes and then connects various nodes according to regular patterns to form several equally sized two-dimensional triangular elements. The area of each triangular element is the same. Considering the small thickness of the bonding interface in actual concrete materials, the thickness of the bonding interface in the meso-structure simulation of concrete is usually taken as between 0.2 and 0.8 mm [24]. In this study, a mesh size of 0.5 mm was used for our calculations. The schematic diagram of this meshing method is shown in Figure 3.

#### 3.3.2. Layered Subdivision Mesh Model of Aggregate

In 1934, the Russian mathematician Delaunay proved that there is only one triangulation method that maximizes the sum of the minimum interior angles of all triangles in a given region. Therefore, the Delaunay triangulation method avoids the appearance of pathological triangles as much as possible and is more suitable for the automatic meshing of finite element meshes. The Delaunay triangulation is the dual graph of the Voronoi diagram [25], and its study is based on the Voronoi diagram.

The Voronoi diagram is composed of a series of polygons *V_i_*. For a given set *X* of *N* discrete points in a plane, *X*, $X=\{X_{1},X_{2},\ldots ,X_{N}\}$, the set of all points *X_i_* whose closest distance is to point *X_i_* is called the Voronoi region $V(X_{i})$ of point *X_i_*. $V(X_{i})$ can be obtained from Equation (10) [25].

(10) $$V(X_{i})=\left\{X|d(X,X_{i})\leq d(X,X_{j}),j=1,2,3,\ldots ,N,j\neq i\right\}$$

**Figure 4:**
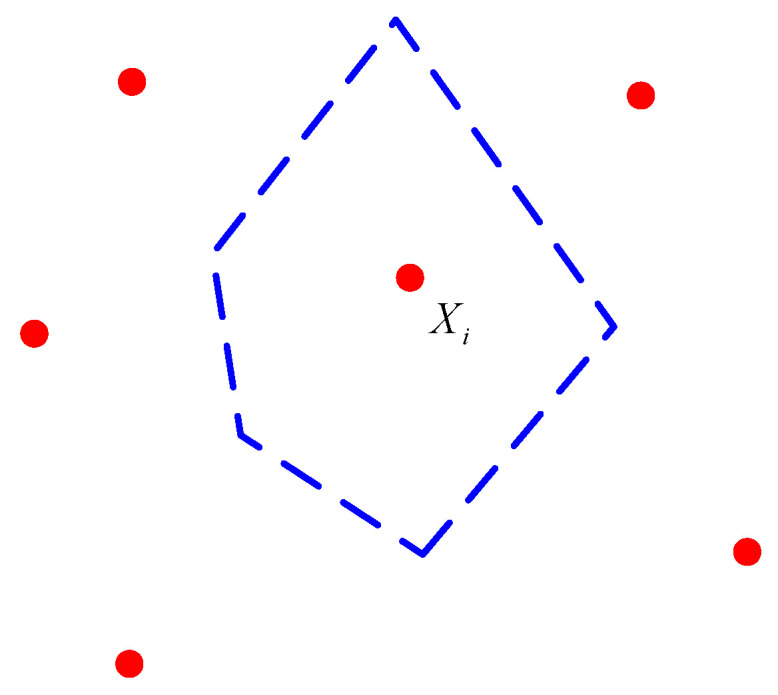
Voronoi region.

**Figure 5:**
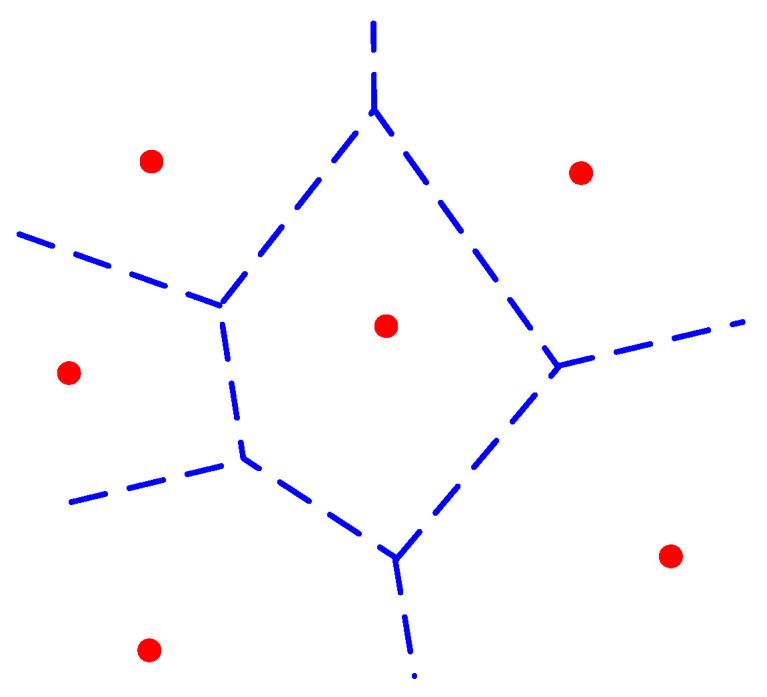
Voronoi diagram.

The Euclidean distance from point *X* to point *X_i_* is represented. Figure 4 shows the Voronoi region $V(X_{i})$ of point *X_i_*, where the *N* points in set *X* have *N* corresponding Voronoi regions. The shape formed by these *N* Voronoi regions is called the Voronoi diagram, as shown in Figure 5.

**Figure 6:**
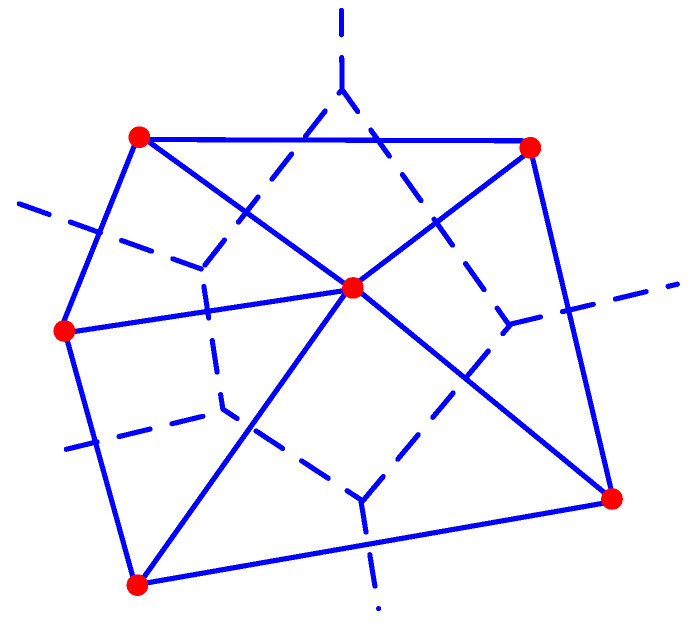
The Delaunay triangulation.

If the center points of each Voronoi region are connected, a series of triangles are formed. This triangulation is called the Delaunay triangulation, represented by solid lines in Figure 6. It is obvious that in two-dimensional space, each edge of a Voronoi region is the perpendicular bisector of two points in set *X*. Therefore, the circumcenter of each triangle coincides with the intersection of the perpendicular bisectors of its three edges.

The Delaunay triangulation has the following characteristics:

i. The Delaunay triangulation is unique.
ii. For a given set of points, the circumcircle of any triangle in the Delaunay triangulation does not contain any other discrete points in the set, and all Delaunay triangles do not overlap.
iii. The smallest interior angle of the triangles obtained from the Delaunay triangulation is the largest. Based on this characteristic, the Delaunay triangulation is the most regular and closest to equilateral triangles.

In summary, the principle of the Delaunay triangulation is not complicated and has some unique advantages. However, implementing the automatic Delaunay triangulation using computer programming is still challenging. This study proposes a new algorithm based on the MATLAB software to implement the automatic Delaunay triangulation of concrete numerical specimens. The specific approach includes the following three points:

i. Point distribution.

This study examines lightweight aggregate concrete as a composite material comprising three phases: lightweight aggregate particles, a cement mortar matrix, and the bond interface layer that exists between the aforementioned. The outermost element of the aggregate is used as the interface transition zone. The point distribution includes a uniform distribution in the cement mortar matrix of the concrete, as well as a regular distribution in the aggregate and bond interface layer to improve the accuracy of element discrimination as much as possible while reducing the computational complexity. To produce uniform points in the concrete matrix, the ‘meshgrid’ command in MATLAB can be used.

Where [*X*, *Y*] = meshgrid (*x*, *y*), with *x* and *y* specifying the range of points in the *x* and *y* directions. *X* and *Y* are the resulting vectors of the point coordinates that meet the target spacing.

To produce scattered points in the circular aggregate and bond interface layer of the concrete, this article uses a self-made function ‘txyqd’ in MATLAB to produce a series of concentric circles inwardly from the circular aggregate with a certain radius difference and distribute points on each circle according to a certain interval to facilitate the subsequent formation of the mesh. Its usage is as in Equation (11) [25].

[*x*1, *y*1, *sL*] = txyqd(*x*, *y*, *r*1, *ms*) (11)

where *x* and *y* are the center coordinates of each concentric circle, *r*1 is the radius, and the variable ‘*ms*’ is the spacing value for point distribution in the concrete matrix. The output is the horizontal and vertical coordinates of the points placed on the circular circumference and the number of points on each circle, where *sL* = round(2*pi*r1/*ms*). It should be noted that the value of ‘*ms*’ should be suitable for the thickness of the bond interface layer. Otherwise, slender triangles may appear excessively.

ii. Perform Delaunay triangulation.

The Delaunay triangulation command is *tri* = Delaunay (*x*, *y*), where the row number of the returned value represents the element number of each triangle, and the three values in each row represent the node numbers of the vertices of each triangle.

iii. Property determination.

**Figure 7:**
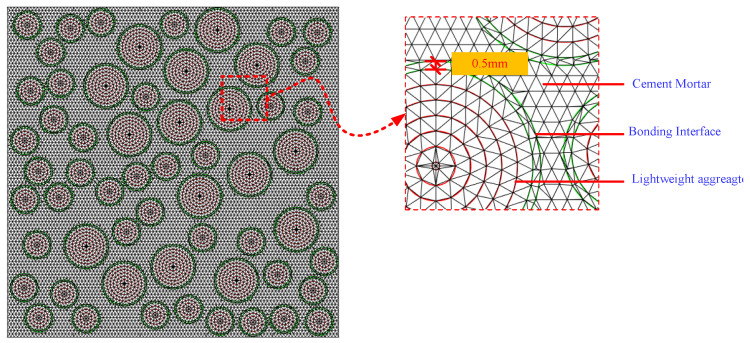
The mesh model of aggregate separated inside and outside (Mesh generation method 2).

After meshing, the mesh is projected onto the random aggregate model region, and material properties are assigned to each element based on the relative positions of its nodes. The element type is determined by judging the relative positions of the nodes of the Delaunay triangles according to the following principles. An element is classified as a lightweight aggregate particle element if all three vertices of its Delaunay triangle are within the coordinate range of the lightweight aggregate particles. An element is classified as a cement mortar element if all three vertices of its Delaunay triangle are within the coordinate range of the cement mortar matrix. Otherwise, the element is classified as an interface element. Figure 7 shows the Delaunay triangulation mesh of a 100 mm × 100 mm lightweight aggregate concrete specimen obtained using the aforementioned method.

Table 1 displays the quantity of mesh elements generated by the two methods. The results indicate that the second method produces merely 32% of the element count achieved by the first method, which can save computational time and improve the efficiency of preprocessing in the finite element numerical calculation process.

### 3.4. Finite Element Meshing of Numerical Specimen Model

The water–cement ratio is a key factor affecting the strength of concrete, mainly affecting the mechanical properties of the cement mortar and bond interface in concrete. The empirical equations for the water–cement ratio and cement mortar and bond interface are summarized by Watson [25], as shown in Equations (12)–(15).

(12) $$c/w=0.047f_{\mathrm{cm}}+0.5$$

(13) $$E_{m}=1000\left(7.7\ln\left(f_{\mathrm{cm}}\right)-5.5\right)$$

(14) $$f_{tm}=1.4\ln\left(f_{c}{}_{m}\right)-1.5$$

(15) $$f_{tITZ}=-1.44w/c+2.3$$

where w/c is the water–cement ratio of the concrete, $f_{cm}$ and $f_{tm}$ are the strength under compression and tension of the cement mortar, $E_{m}$ is the elastic modulus of the cement mortar, and $f_{tITZ}$ is the tensile strength of the bonding interface.

The basic mechanical parameters of lightweight aggregates cannot be measured by standard test methods, such as those for conventional coarse aggregates, making the selection of mechanical parameters difficult. During compressive strength tests of lightweight aggregates, there are large gaps between particles, which are filled with cement mortar with a higher strength in actual lightweight aggregate concrete specimens. Therefore, the compressive strength of the lightweight aggregate particles themselves should be higher than the measured compressive strength. This study integrates ‘GBT 17431.1-2010 Lightweight Aggregates and their test methods Part 1: Lightweight Aggregates’ to determine the strength of lightweight aggregates in numerical simulations. The tensile strength of lightweight aggregate concrete is commonly estimated as approximately 1/10 times the value of its compressive strength based on empirical observations when specific test data are unavailable [26]. The elastic modulus $E_{a}$ of lightweight aggregate can be calculated as shown in Equation (16) [26].

(16) $$E_{a}=0.0106\rho^{2}$$

where ρ is the apparent density of lightweight aggregate particles. The tensile strength of the bonding interface can be determined by Equation (15). This study focuses on lightweight aggregate concrete, where the bond between the cement mortar and aggregate is relatively tight, and the tensile strength can be appropriately increased. According to references [27,28], the elastic modulus of the bonding interface is 40% to 70% of the mortar matrix.

### 3.5. Constitutive Relationship of Material

According to the strain equivalence principle proposed by Lemaitre [29], the nominal stress *σ* of the damaged material can be represented by utilizing the strain of the effective stress in the undamaged material. *σ* can be calculated according to Equation (17) [29].

(17) $$\sigma =E_{0}(1-D)\varepsilon$$

Assuming that damage does not impact the Poisson’s ratio, the elastic modulus *E* following damage can be determined based on the initial elastic modulus. *E* can be calculated according to Equation (18) [29].

(18) $$E=E_{0}(1-D)$$

where $E_{0}$ represents the initial elastic modulus, and D is the damage factor.

Therefore, the damaged elastic modulus of the three-phase medium in lightweight aggregate concrete can be represented as Equation (19) (mortar (m), aggregate (a), and interfacial transition zone (ITZ)).

(19) $$\left\{ \begin{array}{l} E_{a}=E_{a0}(1-D_{a})\\ E_{m}=E_{m0}(1-D_{m})\\ E_{ITZ}=E_{ITZ0}(1-D_{ITZ}) \end{array}\right.$$

Many scholars have concluded that the reason for the nonlinear macroscopic stress–strain curve of concrete under external loads is mainly the expansion of micro cracks [17,18,19]. This study uses a multi-segment damage constitutive model [17,18,19] to describe the damage evolution process of lightweight aggregate concrete. The damage factors of uniaxial tension and uniaxial compression can be obtained by Equations (20) and (21) [17,18,19], respectively.

(20) $$D_{t}=\{\begin{array}{ccc} 0 & & \varepsilon \leq \varepsilon_{t0}\\ 1-\frac{\varepsilon_{t0}}{\varepsilon }+\frac{\varepsilon -\varepsilon_{t0}}{\eta_{t}\varepsilon_{t0}-\varepsilon_{t0}}\frac{\varepsilon_{t0}}{\varepsilon }\left(1-\alpha \right) & & \varepsilon_{t0}<\varepsilon \leq \eta_{t}\varepsilon_{t0}\\ 1-\frac{\alpha }{\xi_{t}-\eta_{t}}\frac{\varepsilon -\eta_{t}\varepsilon_{t0}}{\varepsilon }+\frac{\alpha \varepsilon_{t0}}{\varepsilon } & & \eta_{t}\varepsilon_{t0}<\varepsilon \leq \xi_{t}\varepsilon_{t0}\\ 1 & & \varepsilon >\xi_{t}\varepsilon_{t0} \end{array}$$

(21) $$D_{c}=\{\begin{array}{ccc} 1-\frac{\beta }{\lambda } & & \varepsilon \leq \lambda \varepsilon_{c0}\\ 1-\frac{1-\beta }{1-\lambda }\frac{\varepsilon -\lambda \varepsilon_{c0}}{\varepsilon }-\beta \frac{\varepsilon_{c0}}{\varepsilon } & & \lambda \varepsilon_{c0}<\varepsilon \leq \varepsilon_{c0}\\ 1-\frac{1-\gamma }{1-\eta_{c}}\frac{\varepsilon -\varepsilon_{c0}}{\varepsilon }-\frac{\varepsilon_{c0}}{\varepsilon } & & \varepsilon_{c0}<\varepsilon \leq \eta_{c}\varepsilon_{c0}\\ 1-\frac{\gamma \varepsilon_{c0}}{\varepsilon } & & \eta_{c}\varepsilon_{c0}<\varepsilon \leq \xi_{c}\varepsilon_{c0}\\ 1 & & \varepsilon >\xi_{c}\varepsilon_{c0} \end{array}$$

where $\varepsilon_{0}$ represents the maximum strain, and *η* represents the coefficient of residual strain, which falls within the range of 1 to 5. *ξ* represents the ultimate strain coefficient, where *ξ* is greater than *η*. *λ* denotes the elastic strain coefficient, which takes values between 0 and 1. *ꞵ* represents the coefficient of elastic compressive strength, ranging from 0 to 1. *γ* represents the coefficient of residual compressive strength, and *α* represents the coefficient of residual tensile strength. The subscripts “t” and “c” signify the tension and compression states, respectively.

### 3.6. Discussion of Calculation Results Using Two Different Mesh Generation Methods

To provide a more intuitive demonstration of the higher computational efficiency of the second meshing method proposed in this study, this section establishes a mesoscale model using the proposed two meshing methods. When performing finite element calculations, the two models corresponded completely in terms of size, random aggregate distribution, material parameters, constitutive models, boundary conditions, and loading methods.

#### 3.6.1. Stress–Strain Relationship under Different Meshes

**Figure 8:**
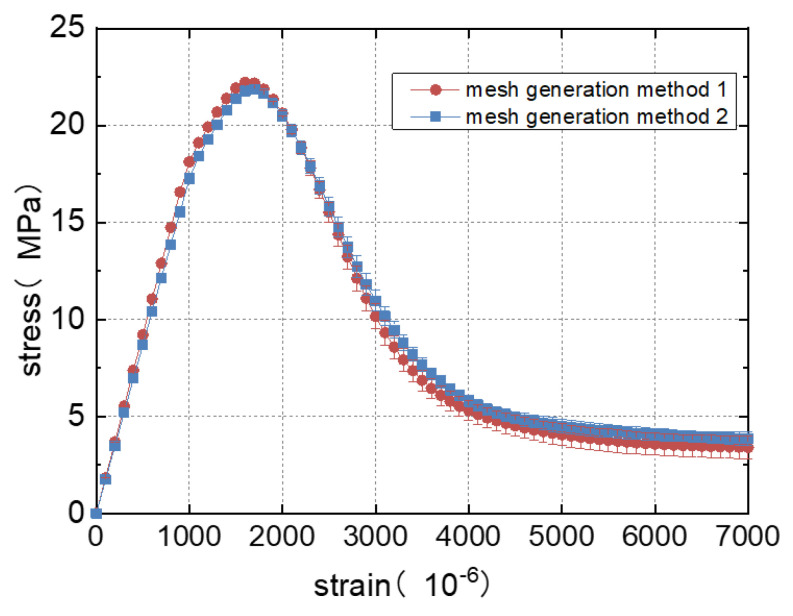
Stress–strain curve of uniaxial compression obtained by different mesh methods.

**Figure 9:**
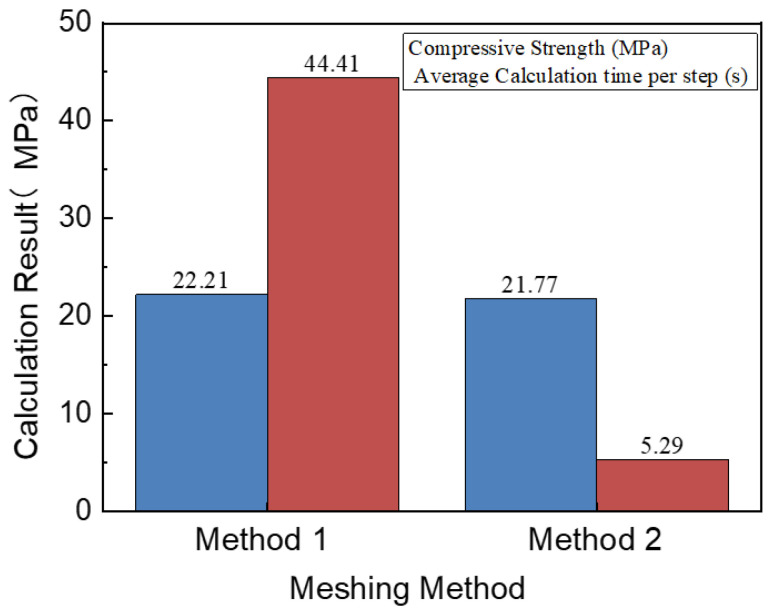
The results obtained by different mesh methods.

The stress–strain curves obtained using these two meshing methods are shown in Figure 8. To eliminate the influence of aggregate distribution, each curve corresponding to each meshing method included the results of calculations for three sets of random aggregate models. It is noted that the stress–strain curves obtained by these two meshing methods exhibit similar trends, with only slight differences in peak stress. The specific calculation results are shown in Figure 9, where the compressive strength represents the average value of the three sets of specimens.

From Figure 8 and Figure 9, it is noted that the compressive strength values calculated by these two meshing methods are similar, and the stress–strain curves are basically the same, so regardless of which meshing method is used, as long as the appropriate mesh size is selected, the calculation error can be controlled.

#### 3.6.2. Computational Efficiency under Different Meshes

From Figure 9, it is obvious that the efficiency of using the two mesh models for calculations is different. When using the uniform meshing method for calculations, only a very small mesh size can be used due to needing to consider the thickness of the bond. Moreover, in this method, it is difficult to control the mesh density of different regions according to the needs, resulting in an increased computational workload and longer calculation time. Meanwhile, the second method has fewer elements and a shorter calculation time. It can be seen that the calculation speed of the second method is 8.4 times that of the first method. Therefore, the second meshing method is used to ensure the efficiency and accuracy of the calculations in this study.

### 3.7. Validation of Numerical Analysis Programs

In order to verify the correctness of the program and algorithm developed in this study, this section takes a two-dimensional specimen with dimensions of 100 mm × 100 mm under both tension and compression as examples, respectively, and performs uniaxial displacement loading. A homogeneous material is used with the following microscale parameters: an elastic modulus of 30 GPa, tensile strength of 3 MPa, compressive strength of 30 MPa, Poisson’s ratio of 0.2, residual tensile strength coefficient of *λ* = 0.1, and residual compressive strength coefficient of α = 0.3, η = 4, and ξ = 10. The multi-line damage constitutive model is used for the compression calculation, and the bi-linear constitutive model is used for the tension calculation. The loading step size is 0.001 mm for the tension and 0.01 mm for the compression.

**Figure 10:**
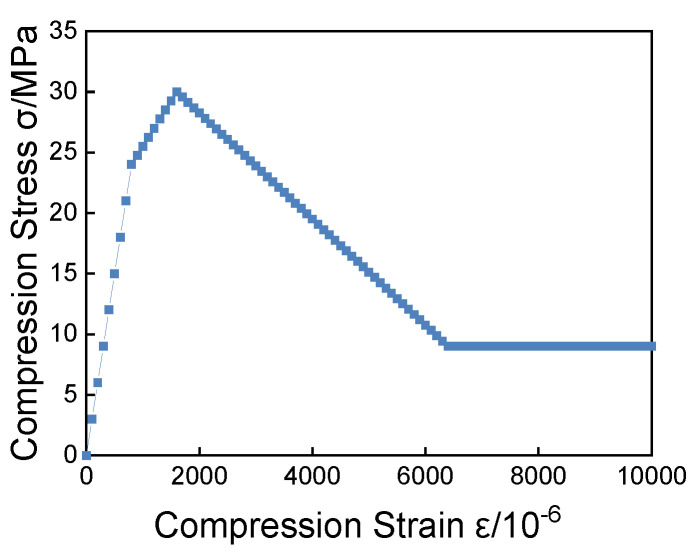
Tensile stress–strain curve.

**Figure 11:**
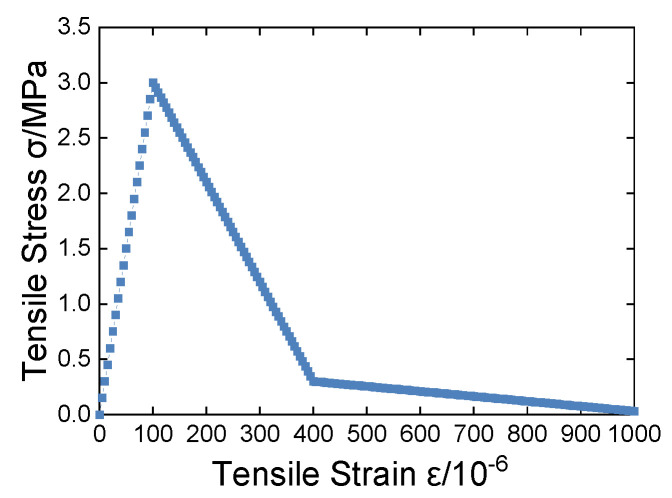
Compressive stress–strain curve.

The comparative analysis between the numerical solution and the theoretical value is as follows:

The calculated results are shown in Figure 10 and Figure 11.

It is noted that the stress–strain curves of the specimen obtained by the nonlinear BFEM program introduced in this study are consistent with the theoretical curves. The analysis result proves the correctness and effectiveness of the program and provides a reliable theoretical basis and simulation conditions for our research.

## 4. Mesoscopic Numerical Simulation of Uniaxial Compression Test

### 4.1. Uniaxial Compression Calculation Model

**Figure 12:**
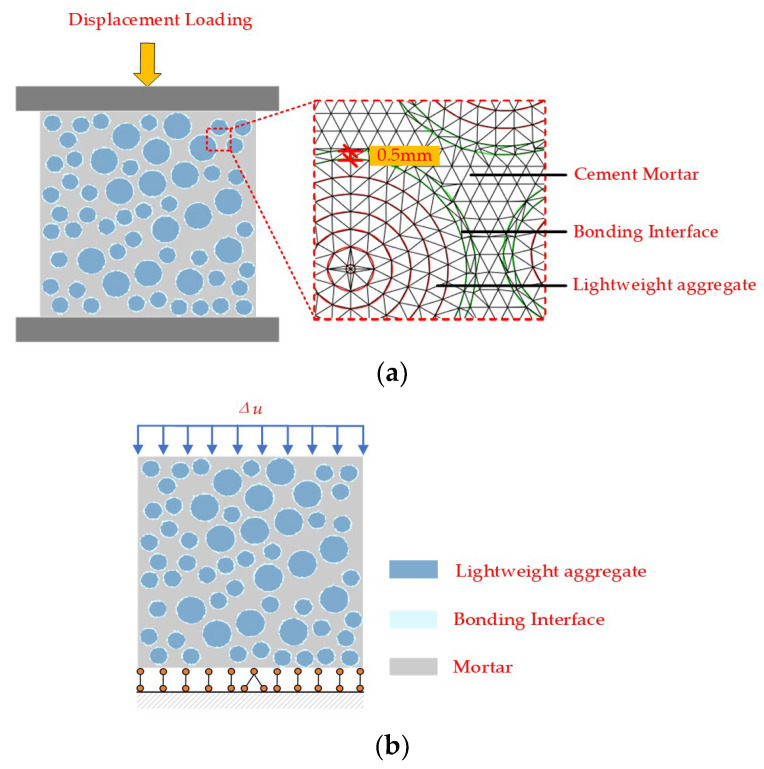
Loading model of uniaxial compression. (**a**) Compression test specimen and meso-structural mesh. (**b**) Displacement loading, displacement boundary conditions, and each phase medium.

The specimen used in this numerical simulation is a cubic specimen with a size of 100 mm × 100 mm × 100 mm, and the calculation model is simplified to a two-dimensional model with a cross-sectional size of 100 mm × 100 mm. The mesh size of mortar and aggregate is 1 mm. The thickness of the bonding interface in the microstructure calculation simulation of concrete is usually between 0.2 and 0.8 mm [24], and this study controls the thickness of the bonding interface, which is set to 0.5 mm. During loading, all nodes on the bottom edge are constrained by vertically displacement, and the central node at the bottom is constrained by horizontal and vertical displacement. Static displacement loading is carried out stepwise along the vertical direction, and the displacement value of each loading step is set to 0.01 mm, as shown in Figure 12.

The coarse aggregates used in the numerical simulation of this study were all between 5 and 15 mm. In order to make the aggregate grading in the model meet the maximum density curve proposed by Fuller [30], the aggregate was divided into two gradations of 5–10 mm and 10–15 mm. According to Equation (4) [23], the proportion of 5–10 mm aggregate can be computed as a percentage by utilizing Equations (22) and (23) [23].

(22) $$P=\frac{P(D<10\mathrm{mm})-P(D<5\mathrm{mm})}{P(D<15\mathrm{mm})-P(D<5\mathrm{mm})}\times 100\%=56.59\%\;10–15\;\mathrm{mm}\;\text{Percentage}\;\text{of}\;\text{aggregate}:$$

(23) $$P=\frac{P(D<15\mathrm{mm})-P(D<10\mathrm{mm})}{P(D<15\mathrm{mm})-P(D<5\mathrm{mm})}\times 100\%=43.41\%$$

Consequently, in the experiment [30], the coarse aggregate used was fly ash ceramic particles produced in Tianjin, with an apparent density of 1227 kg/m^3^ and an element volume usage of 507 kg/m^3^. The proportion of the volume occupied by the coarse aggregate can be determined by Equation (24) [23].

(24) $$P_{k}=\frac{507}{1227}=41.32\%$$

In this numerical simulation, only coarse aggregate particles were used with a volume fraction of 40%. *P_k_* can be calculated as shown in Equations (25)–(27).

(25) $$P_{k}(D<5\mathrm{mm})=0\%$$

(26) $$P_{k}(D<10\mathrm{mm})=0.4\times 56.59\%=22.64\%$$

(27) $$P_{k}(D<15\mathrm{mm})=0.4\times 100\%=40\%$$

The above data are substituted into Equation (5) [23] for calculation, and the coarse aggregate area content of the specimen in the two-dimensional cross-section can be obtained as Equations (28)–(30) when the aggregate volume rate is 40%.

(28) $$P_{c}(D<15\mathrm{mm})=39.92\%$$

(29) $$P_{c}(D<10\mathrm{mm})=19.42\%$$

(30) $$P_{c}(D<5\mathrm{mm})=0\%$$

Then according to Equation (5), the number of $D_{1}<D<D_{2}$ coarse aggregate particles with different particle sizes within different cross-sectional areas can be calculated. The calculation results are shown in Table 2.

According to the empirical equation, the material parameters and constitutive parameters selected for this numerical simulation experiment are shown in Table 3 and Table 4.

### 4.2. Calculation Results of Numerical Model

**Figure 13:**
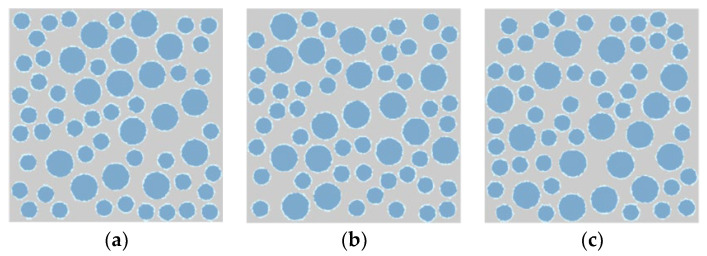
Numerical specimens of lightweight aggregate concrete. (**a**) Specimen 1. (**b**) Specimen 2. (**c**) Specimen 3.

The three numerical specimens of lightweight aggregate concrete used in this simulation were obtained by selecting different random numbers for aggregate placement, as shown in Figure 13. The only difference among the specimens was the distribution of aggregates.

This section utilizes a micro-damage calculation program based on the principle of potential energy and the BFEM. The numerical simulation of uniaxial compression tests was conducted using the three aforementioned randomly generated aggregate specimens, with the criterion for element failure determined by the maximum tensile strain. The calculation results of the three specimens and the experimental data are presented in Table 5. It is noted that the peak stress average values of the three specimens fit well with the experimental data [30] with small errors.

**Figure 14:**
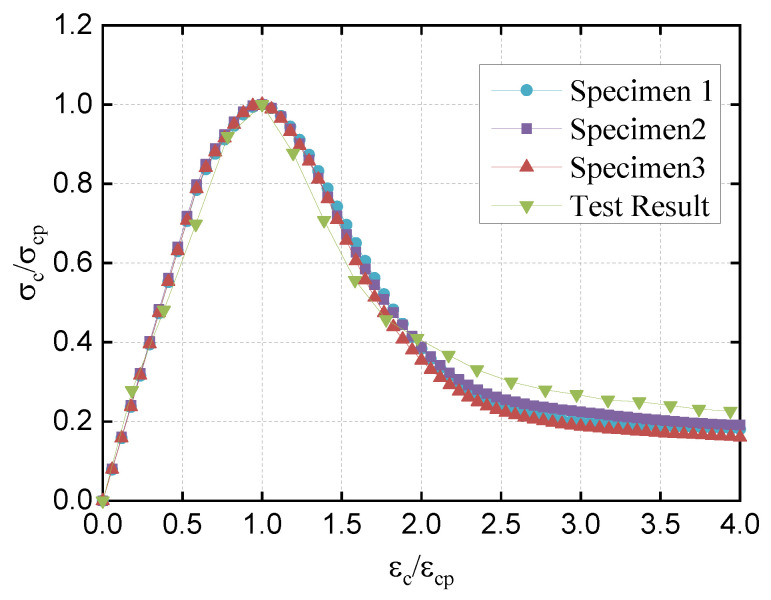
Dimensionless stress–strain curves under uniaxial compression.

Using $\sigma_{c}/\sigma_{cp}$ as the vertical axis and $\varepsilon_{c}/\varepsilon_{cp}$ as the horizontal axis, we plotted the dimensionless stress–strain curve for both the numerical simulation and experiment [30], as shown in Figure 14.

### 4.3. Comparison and Analysis of Results

From Figure 14, it can be seen that, at the beginning of loading, the specimen is basically in an elastic state. As the displacement load increases, the stress value of the specimen increases linearly. When approaching the peak stress, the speed of the increase in stress slows down due to the local elements of the specimen entering the damage state.

After the peak stress point, weaker elements in the specimen start to suffer damage successively, cracks in the specimen expand and penetrate rapidly, and the stress value starts to decrease. Finally, the stress of the specimen tends to be a lower value.

The stress–strain relationship curve obtained by numerical simulation in this study matches well with the experimental results [30], which demonstrates the rationality of the model and the selected parameters in showing the stress rising segment, peak stress point, and corresponding downward segment after damage occurs in the specimen under uniaxial compression.

Additionally, it is noted that the stress–strain curves obtained from the above three randomly generated specimens for uniaxial compression are basically the same, indicating that the stress of lightweight aggregate concrete under uniaxial compression is not significantly affected by the random distribution of aggregates.

**Figure 15:**
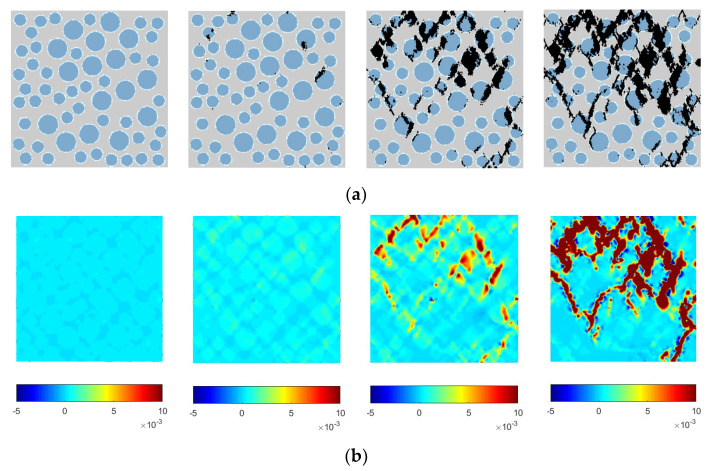

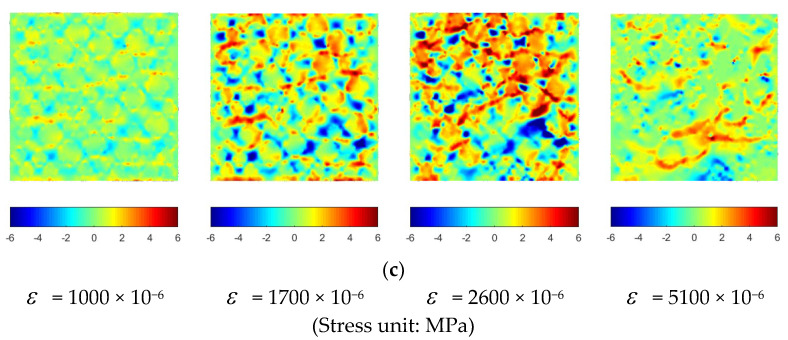
Failure mode, as well as stress and strain contour plots under uniaxial compression. (**a**) Damage and failure diagram of Specimen 1 under uniaxial compression. (**b**) Maximum principal strain contour plots. (**c**) Maximum principal stress contour plots.

To analyze the stress characteristics and damage failure process of lightweight aggregate concrete under uniaxial compression, the calculation results are visualized. Using the graphics display module in the Fortran program, the damage and failure diagram of Specimen 1 under uniaxial compression is drawn. MATLAB-R2022a is used to plot the maximum principal strain contour plots and the maximum principal stress contour plots of the elements during the uniaxial compression process of lightweight aggregate concrete, as shown in Figure 15. In the failure process diagram, the mortar is light gray, the lightweight aggregate is blue, the interface element is light blue, and the failure element is black.

### 4.4. Discussion of the Numerical Results

Based on the damage and failure diagrams and the stress or strain contour plots, the following analysis can be made:

i. Comparing ordinary concrete and recycled concrete [17,18,19], the failure mechanism of lightweight aggregate concrete differs from that of ordinary concrete and recycled concrete. In ordinary concrete, failure occurs when cracks bypass the aggregate particles and propagate along the weaker interface transition zone or mortar matrix. However, in lightweight aggregate concrete, failure mainly occurs due to aggregate cracking, with cracks penetrating throughout the entire specimen.
ii. Initially, the stress and strain distributions in the specimen are relatively uniform. As loading continues, stress concentration phenomena start to appear due to the heterogeneity of lightweight aggregate concrete, for which the mechanical properties of each phase’s medium differ. This phenomenon of the stress concentration is the same as that of ordinary concrete and recycled concrete [17,18,19]. When cracks first appear, they mainly concentrate in the upper and middle parts of the specimen. The elements of the lightweight aggregate and a small amount of the bonded interface are the first to enter the damage stage, as the strength of the lightweight aggregate is lower than that of the other materials, making it the weakest part of the specimen. This is different from the damage of ordinary concrete and recycled concrete [17,18,19].
iii. As the displacement load continues to increase, the stress values of the lightweight aggregate particles and their upper and lower elements become larger, as indicated by the stress contour plots. Cracks in the specimen begin to expand and propagate along the darker-colored areas of the stress concentration zone in the contour plots. In the later stage of loading, the specimen is severely damaged, with numerous cracks appearing inside. These cracks release stress, reducing the stress concentration zone and making the overall stress distribution more uniform, corresponding to the descending segment of the stress–strain curve. This is same from the damage of ordinary concrete and recycled concrete [17,18,19].
iv. By analyzing the damage and failure diagrams and stress or strain contour plots, the complete process of crack appearance and propagation in the specimen during compression was obtained. The characteristics of the stress and strain distribution during compression correspond to the crack distribution in the damage and failure diagrams, validating the correctness of the damage and failure diagrams.

In summary, the shape and characteristics of the stress–strain curve of lightweight aggregate concrete are the external manifestation of changes in the internal element structure. The macroscopic failure of lightweight aggregate concrete is the result of the gradual damage of the internal micro-cracks and the progressive failure of each phase’s material, eventually resulting in a loss of bearing capacity.

## 5. Mesoscopic Numerical Simulation of Uniaxial Tension Test

### 5.1. Uniaxial Tensile Calculation Model

**Figure 16:**
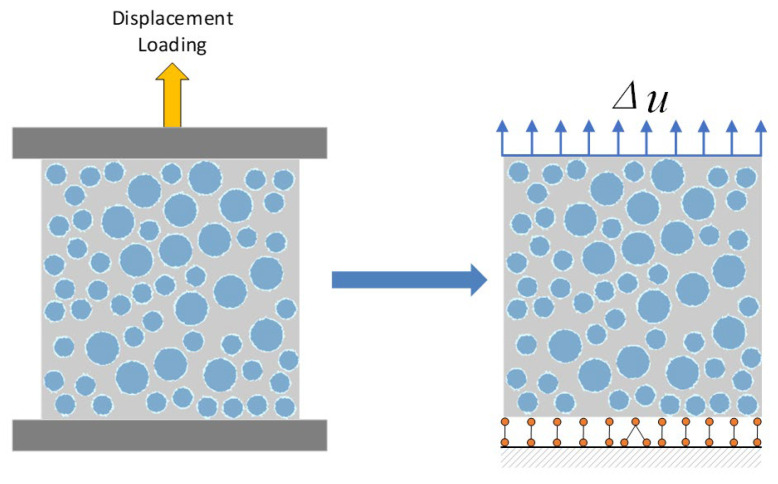
Loading model of uniaxial tensile.

In this numerical simulation, a two-dimensional cross-sectional size of 100 mm × 100 mm was used for the specimen. During loading, all nodes on the bottom edge were vertically constrained, and the center node on the bottom was horizontally and vertically constrained. The static displacement was loaded step by step along the vertical direction, and the displacement value of each loading step was ∆u=0.001 mm, as shown in Figure 16.

### 5.2. Calculation Results of Numerical Model

The three numerical specimens of lightweight aggregate concrete used in this uniaxial tensile simulation are the same as those used in uniaxial compression.

**Figure 17:**
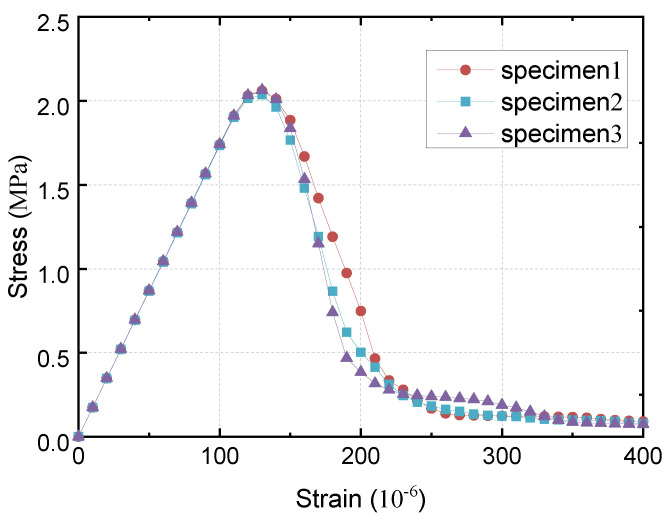
Stress–strain curve of uniaxial tension.

Using the micro-scale damage calculation program based on the principle of potential energy, this section conducts uniaxial tensile tests on the three randomly graded aggregate numerical specimens mentioned above and judges the failure of the element based on the maximum tensile strain criterion. The stress–strain curve of the numerical simulation is plotted with stress as the ordinate and strain as the abscissa, as shown in Figure 17. In the numerical simulation of uniaxial tension, except for the displacement loading value, the other parameters are consistent with those under uniaxial compression.

Using the micro-damage calculation program based on the principle of potential energy, this section conducts uniaxial tensile tests on the above three random aggregate numerical specimens and judges the failure of the element based on the maximum tensile strain criterion. The stress–strain curves of the numerical simulations are plotted with stress as the ordinate and strain as the abscissa, as shown in Figure 17. In the numerical simulation of uniaxial tension, except for the displacement loading value, the other parameters are consistent with those of uniaxial compression.

### 5.3. Analysis of Results

The average peak stress values of the three numerical specimens calculated are 2.05 MPa, and the corresponding strain values are 130 × 10^−6^. The stress–strain curves of the three random aggregate specimens show a similar trend, and the rising section of the curve is approximately a straight line, indicating that the specimens are basically in an elastic state in the initial stage of loading and have not yet produced cracks.

As the displacement load increases, some weak elements in the middle of the specimen begin to get damaged, the rate of the increase in stress slows down, more and more elements are damaged successively, and the specimen enters the softening stage. Afterward, the stress decreases rapidly and eventually goes to zero, which align with the actual situation.

Therefore, the numerical model established in this study and the failure criterion adopted can conduct a finite element calculation and analysis of lightweight aggregate concrete specimens. The method of the BFEM can describe their mechanical behavior under uniaxial tension and obtain the overall stress–strain curve. The results of the BFEM show the rising section, peak stress point, and descending section corresponding to damage and softening.

**Figure 18:**
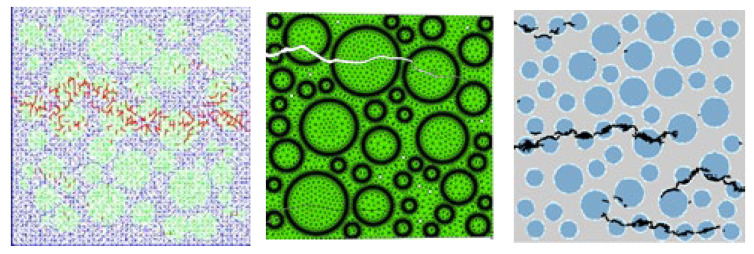
Failure modes of uniaxial tension.

Due to the difficulty of realizing uniaxial tensile tests, there are currently few comparable experimental data. The final failure mode of the lightweight aggregate concrete specimens obtained in this section is listed in Figure 18. It can be seen that the cracks break from the weakest lightweight aggregate particles when the lightweight aggregate concrete specimens are subjected to tensile stress.

**Figure 19:**
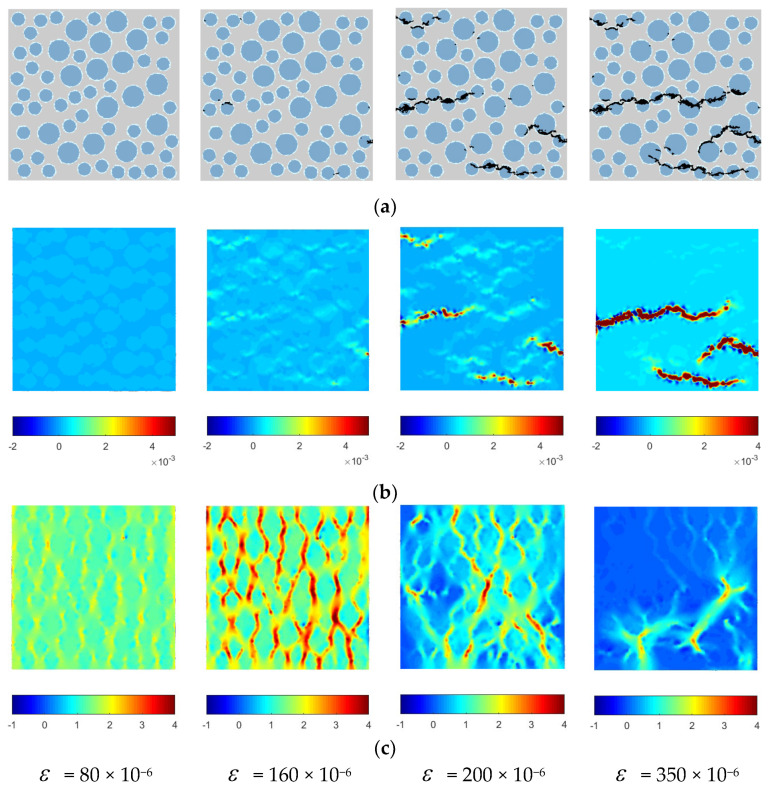
Failure modes, as well as stress and strain contour plots under uniaxial tension. (**a**) Damage and failure diagram of Specimen 1 under uniaxial compression. (**b**) Maximum principal strain contour plots. (**c**) Maximum principal stress contour plots (MPa).

Using the graphic display module in the Fortran program, the damage and failure diagram of the specimen under uniaxial tension was plotted using specimen 1 as an example. The maximum principal strain cloud map and the maximum principal stress contour plots of the elements during the uniaxial tension process of the lightweight aggregate concrete were drawn using MATLAB programming, as shown in Figure 19. In the failure process diagram, the mortar is light gray, the lightweight aggregate is blue, the interface element is light blue, and the failed element is black.

### 5.4. Discussion of the Numerical Results

Combining the failure process diagram and the strain contour plots, the changes in the damage state of the specimen during uniaxial tensile loading can be analyzed to reveal the tensile failure behavior of lightweight aggregate concrete. The following observations can be made:

i. During axial tension of the numerical specimen, as displacement is continuously applied, the local elements of the numerical simulation specimen begin to undergo damage and gradually develop cracks, which then extend to different locations and interconnect until the entire specimen is destroyed. From the shape of the crack extension, it can be seen that the cracks first appear inside the lightweight aggregate and are not uniformly distributed throughout the entire specimen, but are mainly concentrated at the upper and lower ends and middle part of the lightweight aggregate in the numerical specimen. When cracks appear in nearby lightweight aggregates, they penetrate the mortar and interfacial layers, eventually resulting in a main crack that penetrates the entire specimen to reach a failure state. This is consistent with the conclusions of previous studies [1,2,3,4,5,6].
ii. During the early stages of loading, the stress and strain distributions of the specimen are relatively uniform. With the increase in displacement load, the stress of the lightweight aggregate particles at the left and right ends of the specimen is much greater than that of other areas of the lightweight aggregate and cement mortar, and the stress concentration occurs in the same direction as the loading. In the later stages of loading, the damage to the specimen is more serious, and the cracks inside the specimen release stress, resulting in fewer concentrated stress regions and a more uniform stress distribution, which correspond to the descending segment of the stress–strain curve. This is the same as the damage of ordinary concrete and recycled concrete [19].
iii. The extension of cracks during loading shows that the damage to the lightweight aggregate is most severe in the three-phase medium, resulting in the weakening of the local mechanical properties of the surrounding area of the lightweight aggregate, making it easier for the cement mortar in this area to undergo damage. A small amount of bonding interface also undergoes damage, but the cracks do not propagate along the bonding interface layer, but rather penetrate through the lightweight aggregate particles to form a main crack. This suggests that the presence of the bonding interface has little effect on the crack extension behavior of the lightweight aggregate concrete specimen, which significantly differs from that of ordinary aggregate and recycled aggregate concrete specimens [19].
iv. The strain distribution during the numerical simulation of the tensile process is completely consistent with the crack distribution in the damage and failure map, which verifies the correctness of the damage and failure map.

## 6. Conclusions

In this study, an automatic dissection algorithm of lightweight aggregate based on the Delaunay triangular mesh algorithm is proposed. The damage analysis based on the BFEM and the corresponding calculation analysis program can numerically simulate the uniaxial tension and compression tests of lightweight aggregate concrete. Our self-developed post-processing software can obtain the stress–strain curve, micro-damage failure process diagram, maximum principal stress contour plots, and maximum principal strain cloud map during loading.

i. The corresponding automatic dissection program can be developed by stratifying the internal area of the lightweight aggregate. This method can effectively improve the efficiency of pre-processing in finite element calculation.
ii. The numerical analysis results in this paper are in good agreement with the experimental results. This proves the effectiveness of the proposed algorithm.
iii. The study on lightweight aggregate concrete shows that the failure mode of lightweight aggregate concrete significantly differs from that of ordinary concrete. It is found that the light aggregate of light aggregate concrete is damaged first in the loading process, and the cracks are broken inside the light aggregate. This indicates that the light aggregate is a weaker area of light aggregate concrete.
iv. The research results provide effective analysis methods and data for engineering applications of lightweight aggregate concrete materials.

In the future, in-depth and systematic numerical research works will be carried out on the failure mechanism of three-point curved beams, the application of image processing technology, the mechanical properties of high-strength lightweight aggregate concrete, and the failure mechanism of fiber lightweight aggregate concrete.

## Figures and Tables

**Table 1:** The number of elements in both mesh models.

Meshing Method	Meshing Method 1	Meshing Method 2
Number of elements	80,000	25,672

**Table 2:** Number of aggregate particles.

Size of Aggregate Particles	10–15 mm	5–10 mm
Number of aggregate particles	16	43

**Table 3:** Material parameters.

Parameters	Compressive Strength/MPa	Tensile Strength/MPa	Elastic Modulus/MPa	Poisson’s Ratio
Lightweight aggregates	16.6	1.66	14,000	0.22
Mortar	46.8	3.88	24,000	0.2
Interface	22.0	2.20	10,000	0.2

**Table 4:** Constitutive curve shape parameters.

Parameter	Mortar	Lightweight Aggregate	Interface
β	0.8	0.85	0.9
γ	0.35	0.4	0.35
α	0.2	0.2	0.2
λ	0.5	0.45	0.5
$\eta_{c}$	3	3	3
$\eta_{t}$	2	3	2
$\xi_{t}$, $\xi_{c}$	10	10	10

**Table 5:** Peak stress of lightweight aggregate concrete specimens.

	Specimen1	Specimen2	Specimen3	Average Value	Experimental Data Results [30]	Error
Peak stress (MPa)	22.03	21.57	21.98	21.86	21.90	0.18%

## Data Availability

The data that support the findings of this study are available from the corresponding author upon reasonable request.
